# Teriparatide and stress fracture healing in young adults (RETURN – Research on Efficacy of Teriparatide Use in the Return of recruits to Normal duty): study protocol for a randomised controlled trial

**DOI:** 10.1186/s13063-021-05556-3

**Published:** 2021-08-30

**Authors:** Alexander T. Carswell, Katharine G. Eastman, Anna Casey, Matthew Hammond, Lee Shepstone, Estelle Payerne, Andoni P. Toms, James W. MacKay, Ann Marie Swart, Julie P. Greeves, William D. Fraser

**Affiliations:** 1 Norwich Medical School, Faculty of Medicine and Health Sciences, University of East Anglia, Norwich, NR4 7TJ UK; 2 Army Health and Performance Research, British Army Headquarters, Ministry of Defence, Andover, SP11 8HT UK; 3 Norwich Clinical Trials Unit, Norwich Medical School, University of East Anglia, Norwich, NR4 7TJ UK; 4 Departments of Endocrinology and Clinical Biochemistry, Norfolk and Norwich University Hospitals NHS Foundation Trust, Norwich, NR4 7UY UK

**Keywords:** Stress fracture, Bone, Musculoskeletal injury, Teriparatide, Parathyroid hormone, Magnetic resonance imaging, Prospective randomised controlled trial, Military, Rehabilitation, Intention-to-treat

## Abstract

**Background:**

Stress fractures are a common and potentially debilitating overuse injury to bone and occur frequently among military recruits and athletes. Recovery from a lower body stress fracture typically requires several weeks of physical rehabilitation. Teriparatide, a recombinant form of the bioactive portion of parathyroid hormone (1–34 amino acids), is used to treat osteoporosis, prevent osteoporotic fractures, and enhance fracture healing due to its net anabolic effect on bone. The study aim is to investigate the effect of teriparatide on stress fracture healing in young, otherwise healthy adults undergoing military training.

**Methods:**

In a two-arm, parallel, prospective, randomised controlled, intention-to-treat trial, Army recruits (*n* = 136 men and women, 18–40 years) with a magnetic resonance imaging (MRI) diagnosed lower body stress fracture (pelvic girdle, sacrum, coccyx, or lower limb) will be randomised to receive either usual Army standard care, or teriparatide and usual Army standard care. Teriparatide will be self-administered by subcutaneous injections (20 μg/day) for 16 weeks, continuing to 24 weeks where a fracture remains unhealed at week 16. The primary outcome will be the improvement in radiological healing by two grades or more, or reduction to grade zero, 8 weeks after randomisation, assessed using Fredericson grading of MRI by radiologists blind to the randomisation. Secondary outcomes will be time to radiological healing, assessed by MRI at 8, 10, 12, 14, 16, 20 and 24 weeks, until healed; time to clinical healing, assessed using a clinical severity score of injury signs and symptoms; time to discharge from Army physical rehabilitation; pain, assessed by visual analogue scale; health-related quality of life, using the Short Form (36) Health Survey; and adverse events. Exploratory outcomes will include blood and urine biochemistry; bone density and morphology assessed using dual-energy X-ray absorptiometry, peripheral quantitative computed tomography (pQCT), and high-resolution pQCT; physical activity measured using accelerometers; and long-term future fracture rate.

**Discussion:**

This study will evaluate whether teriparatide, in addition to standard care, is more effective for stress fracture healing than standard care alone in Army recruits who have sustained a lower body stress fracture.

**Trial registration:**

ClinicalTrials.govNCT04196855. Registered on 12 December 2019.

## Administrative information

The order of the items has been modified to group similar items (see http://www.equator-network.org/reporting-guidelines/spirit-2013-statement-defining-standard-protocol-items-for-clinical-trials/).

**Table:** 

Title {1}	Teriparatide and stress fracture healing in young adults (RETURN – Research on Efficacy of Teriparatide Use in the Return of recruits to Normal duty): study protocol for a randomised controlled trial
Trial registration {2a and 2b}	ClinicalTrials.gov, NCT04196855. Registered 12^th^ December 2019.
Protocol version {3}	Version 1.6, 10^th^ September 2020.
Funding {4}	Fully funded by the Ministry of Defence. Gedeon Richter (Budapest, Hungary) provide teriparatide (Terrosa).
Author details {5a}	^1^Norwich Medical School, Faculty of Medicine and Health Sciences, University of East Anglia, Norwich, NR4 7TJ, UK. ^2^Army Health and Performance Research, British Army Headquarters, Ministry of Defence, Andover, SP11 8HT, UK. ^3^Norwich Clinical Trials Unit, Norwich Medical School, University of East Anglia, Norwich, NR4 7TJ, UK. ^4^Departments of Endocrinology and Clinical Biochemistry, Norfolk and Norwich University Hospitals NHS Foundation Trust, Norwich, NR4 7UY, UK.
Name and contact information for the trial sponsor {5b}	Julie Dawson, Norfolk and Norwich University Hospitals NHS Foundation Trust, Norwich, NR4 7UY, UK.
Role of sponsor {5c}	Norfolk and Norwich University Hospitals NHS Foundation Trust is the trial Sponsor and has delegated responsibility for the overall management of the study to the Chief Investigator and Norwich Clinical Trials Unit. The Ministry of Defence assisted with the study design; identification and access to military Units, potential participants and experimental facilities; and will assist with the strategic management of the trial, interpretation of data and manuscript writing; but will not have ultimate authority over these activities. Manuscripts will require permission to publish from the Ministry of Defence, but this will not be influenced in any way by the outcomes of the trial or direction of effect.

## Introduction

### Background and rationale {6a}

Stress fractures are a common and potentially debilitating overuse injury to bone and are frequently reported among military recruits and athletes [1–6]. Bone normally remodels through a continual, balanced cycle of osteoclastic resorption and osteoblastic bone formation. Sudden increases in training volume of high-intensity, repetitive impact activities (e.g. running and marching) without adequate time for recovery can cause an imbalance in bone remodelling [7], with greater osteoclastic activity at the sites of mechanical loading causing a weakening of the bone, microdamage, and a subsequent stress fracture [5, 8]. Stress fracture injuries can lead to a full fracture without a significant reduction in physical activity levels and a period of rehabilitation [9]. Lower body (i.e. pelvic girdle, sacrum, coccyx, and lower limb) stress fractures require the longest rehabilitation times of all musculoskeletal injuries sustained during Army Infantry training, often exceeding 80 days [2]. The return to training of athletes who have sustained lower body stress fractures takes between 61 and 153 days, on average [6].

Daily subcutaneous injections of teriparatide, a recombinant form of the bioactive portion of parathyroid hormone, containing the first 34 amino acids, have been used to treat osteoporosis, prevent osteoporotic fractures and enhance fracture healing due to its net anabolic effect on bone [10–12]. Continuous infusion of teriparatide has a catabolic effect on bone, whereas intermittent daily injections have an anabolic effect [13–17]. Accelerated bone remodelling, earlier replacement of woven bone with lamellar bone, and improvements in mechanical strength, bone mineral content, and callus volume and strength have been documented after the administration of teriparatide in animal models of fracture healing (e.g. long bones of rat, mouse, rabbit, monkey [10]). A number of studies conducted in postmenopausal women and osteoporotic patients have demonstrated increased bone mineral density [18–21], bone mineral content [20–22], and an increase in biochemical markers of bone formation and resorption [18, 19, 23] following treatment with teriparatide. Pain in limb (reported by ≥ 10% of patients), followed by nausea, headache, and dizziness (each reported by ≥ 1% to < 10% of patients) are the most common adverse reactions to teriparatide [24]; nevertheless, treatment with teriparatide in humans is considered safe [24–26]. In randomised controlled trials, 8 weeks of daily teriparatide accelerated fracture healing in postmenopausal women [27], and parathyroid hormone [1-84] accelerated fracture healing and reduced pain after 8 weeks, and improved mobility in elderly osteoporotic women (aged > 70 years) [28]. No effect of teriparatide on fracture healing or pain was observed in two randomised controlled trials treating men and postmenopausal women (aged ≥ 50 years) [29, 30], but the treatment was limited to 4 weeks [29], and the trials were underpowered [29, 30]. Randomised controlled trials comparing teriparatide with bisphosphonates reported increased bone mineral density and decreased fracture risk with teriparatide use in postmenopausal osteoporosis patients [31], but variable effects on radiological healing, pain, and quality of life have been observed in men and women [32, 33]. The assessment of different outcome measures and inclusion of various fracture types might be responsible for these variable responses to teriparatide compared with bisphosphonates. Teriparatide reduced the risk of future fractures compared with placebo in postmenopausal women with prior fractures [20, 34], but no studies have examined this effect in young adults.

Only one published randomised controlled pilot study has examined the effect of teriparatide on stress fracture healing in young adults (aged 21–45 years) [35]. Using Fredericson grading of magnetic resonance imaging (MRI) to assess the healing of lower limb fractures, four women (67%) who received teriparatide for 8 weeks, compared with three women (43%) in the placebo control group, achieved a two grades or more improvement in healing or reduction to grade zero. Biochemical markers of bone formation increased more in women who received teriparatide compared with placebo [35], indicating a significant anabolic window, whereby teriparatide initially stimulates bone formation, with a rapid increase in bone formation markers during the initial weeks, followed by increases in bone resorption markers in later weeks [36]. A number of case studies of young and elderly adults (32–91 years) support the efficacy of teriparatide for accelerating fracture [10] and stress fracture healing [37]. Despite these encouraging findings, the effectiveness of teriparatide for promoting fracture healing remains unclear, particularly among young, otherwise healthy adults. High quality randomised controlled trials are required to test the efficacy of teriparatide for stress fracture healing.

Optimising the healing of stress fractures is relevant to the military training environment because quality of healing, time to fracture union and return to training can have significant consequences for military recruits in terms of career progression, and risk of re-injury and medical discharge, and the Ministry of Defence in terms of the cost of working days lost in rehabilitation and loss of recruits from training. Changes to initial military training intended to reduce the risk of musculoskeletal injury have mostly been ineffective [2]. Effective strategies for optimising the rehabilitation of stress fracture injuries and expediting a return to training will improve the overall health and wellbeing of individuals and may also reduce medical attrition rates. The aim of the present study is to evaluate the effect of daily teriparatide injections on lower body stress fracture healing in young, otherwise healthy adults undergoing Army training.

### Objectives {7}

The primary objective of the RETURN trial is to investigate the effect of teriparatide in addition to usual Army standard care on lower body stress fracture healing in military recruits, compared with usual Army standard care alone. The primary outcome will be the improvement in radiological healing, assessed using Fredericson grading of MRI, by two grades or more, or reduction to grade zero, 8 weeks after randomisation. Secondary objectives are to examine the effect of teriparatide on (i) time to radiological healing; (ii) time to clinical healing, assessed by clinical severity score calculated from injury signs and symptoms; (iii) time to discharge from Army physical rehabilitation; (iv) pain symptoms; (v) health-related quality of life; and (vi) adverse events. It is hypothesised that treatment with teriparatide will accelerate (i) the rate of radiological healing of lower body stress fractures; (ii) the cessation of pain associated with the injury; and reduce (iii) the time taken to return to Army training, compared with standard care alone. The effect of teriparatide on the following exploratory outcomes will also be investigated: (i) blood and urine biochemistry; (ii) bone density and morphology; (iii) physical activity levels; and (iv) long-term future fracture rate.

### Trial design {8}

**Fig. 1:**
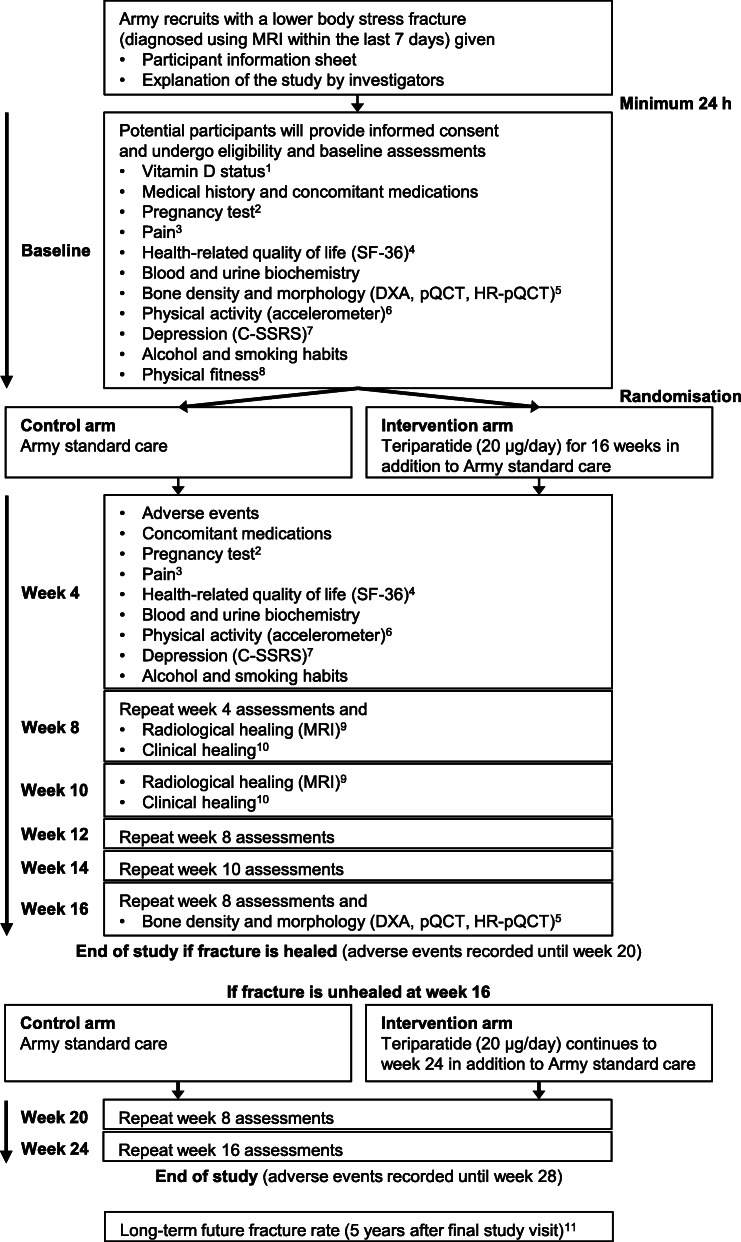
Overview of the trial design, interventions and assessments. ^1^Serum 25-hydroxyvitamin D < 30 nmol/L, deficient; ≥30 nmol/L, not deficient; ^2^Pregnancy test for all at baseline, week 16 and 24, and four-weekly for participants allocated to the intervention arm; ^3^Weekly visual analogue scale; ^4^Short Form (36) Health Survey; ^5^Dual-energy X-ray absorptiometry (DXA) scan of the lumbar spine and hips (baseline and week 16 only); peripheral quantitative computed tomography (pQCT) scan of the diaphyseal tibia; and high-resolution pQCT (HR-pQCT) scan of the metaphyseal tibia and radius; ^6^Accelerometer worn 24 h/day and replaced four-weekly; ^7^Columbia-Suicide Severity Rating Scale (C-SSRS) for all at baseline, and four-weekly for participants allocated to the intervention arm; ^8^Review of Army fitness records; ^9^Magnetic resonance imaging (MRI) scans to cease when fracture is radiologically healed; ^10^Clinical assessments twice weekly after fracture is radiologically healed, and to cease when fracture is clinically healed; ^11^Review of Army medical records

The RETURN trial is a phase III, multicentre, open label, two-arm parallel, prospective, randomised controlled trial (Fig. 1). A 1:1 allocation ratio will be used in this intention-to-treat, superiority trial.

## Methods: Participants, interventions and outcomes

### Study setting {9}

The study will take place in the UK, at the Infantry Training Centre, Catterick, North Yorkshire and a nearby National Health Service (NHS) institution within the County Durham and Darlington NHS Foundation Trust, neither of which are part of the University of East Anglia.

### Eligibility criteria {10}

Men and women, aged 18–40 years, with single or multiple lower body stress fractures, diagnosed by MRI a maximum of 7 days before date of consent, will be eligible to participate. Potential participants must be undergoing Phase One or Two Army training (i.e. initial or trade training) and will, therefore, have recently passed a physician-screened medical assessment. Eligible participants must provide written informed consent and be able to comply with the study protocol, adhere to study visit requirements, and have baseline safety blood test results within adult reference ranges (analytes listed in Table 1).

**Table 1:** Blood and urine biochemistry

		Baseline only	Baseline and weeks 4, 8, 12, 16, 20 and 24
**Blood**	**Safety**	Haemoglobin Platelets White blood cell count Absolute neutrophil count Urate Albumin Bilirubin Alanine aminotransferase Alkaline phosphatase Glucose Parathyroid hormone	Calcium Phosphate Magnesium Sodium Potassium Urea Creatinine High- and low-density lipoproteins Total cholesterol
	**Research**		Parathyroid hormone (including 1-34 and other fragments) Cyclic adenosine monophosphate Vitamin D metabolites Bone remodelling compounds: Carboxyl-terminal collagen cross-links Procollagen type 1 N-terminal propeptide Sclerostin Osteoprotegerin Receptor activator of nuclear factor kappa-B ligand Dickkopf-related protein 1 Fibroblast growth factor 23 Butyrate
**Urine**	**Safety**		Calcium Phosphate
	**Research**		Pyridinoline Deoxypyridinoline Oxalate Cyclic adenosine monophosphate

Additional bone remodelling compounds may be analysed if assay methodologies are validated during the trial

Potential participants will be excluded if they have any of the following: known hypersensitivity to active parathyroid hormone or any excipients listed in the summary of product characteristics [24]; pre-existing hypercalcaemia; a history of skeletal malignancies or bone metastases; any contraindications that would prevent them from undergoing an MRI scan; severe renal impairment (participants with a moderate renal impairment will be treated with caution at the Principal Investigator’s discretion and in accordance with the summary of product characteristics [24]); metabolic bone diseases, including a pre-existing diagnosis of hyperparathyroidism or Paget’s disease of bone; an unexplained elevation of alkaline phosphatase; prior external beam or implant radiation therapy to the skeleton; depression, identified by the Columbia-Suicide Severity Rating Scale (C-SSRS); or are receiving digoxin or any other concurrent therapy that would interfere with the evaluation of the safety or efficacy of teriparatide; currently pregnant, suspect they are pregnant, or are breastfeeding; present with open epiphyses in their diagnostic MRI scan; or are participating in a concurrent drug trial. Potential participants will be excluded if they are in receipt of an investigational drug or biological agent within the 4-week period prior to study entry (or five times the half-life if this is longer). Participants can be entered into other observational studies given prior agreement from the Trial Management Groups (TMGs) of both studies. Women must have a negative serum pregnancy test at baseline and agree to use a highly effective form of contraception (with a failure rate < 1% per year) for the duration of their participation in the study.

Principal Investigators (or medically trained delegates) will be appropriately qualified and licensed to practice within their trial site. Suitably trained staff will undertake delegated trial activities. All investigators will sign an investigator statement to confirm they will comply with the study protocol. Sites responsible for prescribing and dispensing drugs will have a pharmacy that is able to store, prepare and dispense drugs appropriately.

### Who will take informed consent? {26a}

Written informed consent will be obtained by an appropriately trained clinician or delegated member of staff at the NHS institution in person, or remotely (consent form completed by the participant during a telephone or video call) if NHS hospital visits are restricted for any reason. Potential participants will be provided with a participant information sheet and will be invited to meet investigators for a detailed explanation of the study’s aims, methods, benefits and potential hazards. Consent will be obtained a minimum of 24 h after the potential participant receives the participant information sheet and detailed explanation of the study; and a maximum of 7 days after their diagnosis with a lower body stress fracture using MRI. Consent will be obtained in the absence of the participants’ Army Chain of Command, and it will be explained to potential participants that choosing to take part, or not, or choosing to withdraw from the study at any point, will have no bearing on their future Army training or career. Potential participants who wish to take part will provide informed consent before any trial-specific procedures are undertaken. Participants will be invited to provide additional consent to wear an accelerometer for the duration of their participation in the study, however, this is optional, and participants may refuse whilst remaining eligible to take part.

### Additional consent provisions for collection and use of participant data and biological specimens {26b}

Participants will be asked to consent to their samples being stored securely in a pseudoanonymised form for use in future studies, pending ethical approval. No ancillary studies are planned.

## Interventions

### Explanation for the choice of comparators {6b}

There are no current pharmacological therapies indicated for the treatment of stress fractures. The control arm will receive usual Army standard care. Participants will commence a period of physical rehabilitation under the supervision of a physiotherapist and Army exercise rehabilitation instructor, in accordance with standard Army procedures. The intervention arm will receive 20 μg/day of teriparatide by subcutaneous injection in addition to usual Army standard care. This is the only licensed dose for teriparatide in Europe. Teriparatide is licensed for the treatment of osteoporosis in postmenopausal women and in men at increased risk of fracture, and the treatment of osteoporosis associated with sustained systemic glucocorticoid therapy in women and men at increased risk for fracture [24]. An open label design was chosen for a number of reasons: (i) the aims of the study and objective primary endpoint indicated an open label design; (ii) it was not possible to procure a matched placebo; (iii) there will be no requirement for control arm participants to administer themselves with daily injections of placebo for up to 24 weeks, thereby reducing the burden on them, and avoiding the possible ethical implications of requiring them to do so; and (iv) adherence to the injection regime may be improved with intervention arm participants knowing they are receiving active drug. The practical issue of adherence to the injection regime by military recruits, knowing they are receiving the drug, is of importance to the Ministry of Defence and the future roll-out of any treatment regime.

### Intervention description {11a}

All participants will receive usual Army standard care. According to standard Army procedures, all recruits will be taken out of training on confirmation of their injury and referred for extended rehabilitation in a rehabilitation platoon. Recruits may be sent on sick leave, with the duration determined according to the severity of their injury and symptoms. Early rehabilitation will concentrate on upper body, non-impact cardiovascular work and swimming/deep water running. Exercise interventions will aim to maintain cardiovascular fitness using non-impact activities and to maintain strength as pain allows. Once rehabilitation is complete and a recruit is fully fit, they will be graded as fit to return to training. The final functional test before a decision to return to training is made will be the successful completion of a weighted distance march without symptoms, at a level appropriate to the week of training they are returning to. A review by an Army exercise rehabilitation instructor, physiotherapist and general practitioner will be required to determine if a recruit is ready to return to training. The rehabilitation team will be informed of a recruit’s participation in the study, but this will not affect their treatment or rehabilitation programme in any way.

Participants randomly allocated to the intervention arm will receive teriparatide (Terrosa, Gedeon Richter, Budapest, Hungary) in addition to usual Army standard care. Daily subcutaneous injections of teriparatide (20 μg) will be self-administered into the abdomen or thigh using an injection pen and sterile, single use needles. Cartridges of 2.4 mL of a solution containing 600 μg of teriparatide (250 μg/mL) will provide 28 individual fixed doses of 20 μg of teriparatide (20 μg/80 μL solution) when used with a bespoke injection pen. A sharps bin will be provided for the disposal of used needles. Participants will be instructed to store their teriparatide cartridge and injection pen in a refrigerator (2–8 °C). Doses will be reconciled, and a replacement cartridge will be dispensed by the NHS pharmacy every 4 weeks. Daily teriparatide injections will be self-administered for 16 weeks, but where a fracture remains unhealed at week 16, daily injections will continue until week 24. Participants will be given a user manual that fully describes how to use the injection pen and will be trained to use the proper injection technique by investigators. Participants will be instructed to inject at the same time every day. If they forget to inject or are unable to inject at their usual time, participants may take their dose up to 12 h later than planned. When participants are onsite at Infantry Training Centre, Catterick, their teriparatide cartridge and injection pen will be stored by investigators in a temperature monitored refrigerator. When participants are at home there will be no formal temperature monitoring. Participants will be provided with a travel cool pack to transport their teriparatide cartridge and injection pen when travelling between locations.

Participants identified with vitamin D deficiency (serum 25-hydroxyvitamin D < 30 nmol/L [38]) will be treated in accordance with the Ministry of Defence regulation JSP 950, Part 1, Leaflet 2-9-3, irrespective of which arm they have been allocated to. Under the terms of this regulation, to manage vitamin D deficiency, a daily dose of 3200 IU cholecalciferol using cholecalciferol 800 IU (Fultium D_3_/Desunin), or a weekly dose of 60,000 IU (or 20,000 IU on alternate days) using cholecalciferol 20,000 IU capsules will lead to restoration of body stores over 8 to 12 weeks. Long-term maintenance/supplementary therapy is then needed with the equivalent of cholecalciferol 1000 or 2000 IU daily or 10,000 IU weekly.

### Criteria for discontinuing or modifying allocated interventions {11b}

Participants may stop their treatment with teriparatide at any time if they wish. For safety reasons, treatment will be discontinued for individual participants if they experience an unacceptable adverse event or treatment toxicity; an intercurrent illness that prevents further treatment; any change in their condition that in the clinician’s opinion justifies ceasing treatment; or any contraindication to continuing treatment within the summary of product characteristics, including pregnancy [24]. Participants who have their treatment interrupted for 2 weeks or more, intentionally overdose, or exhibit signs of depression (indicated by the C-SSRS completed every 4 weeks prior to teriparatide re-dispensing) will also have their treatment discontinued. Discontinuation of treatment with teriparatide will not be a reason for withdrawal from the study, and participants will complete follow-up visits provided they are willing. Where pre-dose serum calcium is found to be above the upper limit of normal, treatment will be withheld until serum calcium returns to normal and restarted every other day, or every third day according to the clinical judgement of the treating clinician and Chief Investigator. Before restarting treatment, the participant’s correct use of the injection pen will be verified, and differential diagnoses will have been excluded (e.g. primary hyperparathyroidism, excess vitamin D and malignancy). Where pre-dose serum calcium is < 2 mmol/L, treatment will continue with the addition of twice daily calcium (1500 mg) and vitamin D (400 IU) supplementation.

### Strategies to improve adherence to interventions {11c}

Face-to-face reminders about how and when to inject teriparatide, how to store teriparatide, and what to do in the event of a missed dose will take place when the drug is initially dispensed, each time the drug is re-dispensed, and during each study visit. Participants will record self-administration of teriparatide in a daily diary. Diaries will be reviewed by investigators every 4 weeks, alongside any problems that participants might be having with injections, and the reasons for any missed doses. Used teriparatide cartridges will be collected from participants every 4 weeks, with the number of unused doses recorded to monitor adherence to the intervention.

### Relevant concomitant care permitted or prohibited during the trial {11d}

The use of medication bought over the counter or prescribed by other healthcare professionals will be permitted, except for non-steroidal anti-inflammatory drugs and vitamin D or its analogues. Participants’ use of analgesics will be recorded throughout the study using their participant diary. The combined use of teriparatide and cardiac glycosides (e.g. digoxin or digitoxin) may predispose participants to digitalis toxicity [24]; thus, participants required to begin taking cardiac glycosides will be withdrawn from the study. Participants required to begin taking medications that affect serum calcium (including but not limited to lithium, thiazides or alendronic acid) will also be withdrawn from the study.

### Provisions for post-trial care {30}

There will be no provisions for post-trial care, including continuation of treatment with teriparatide. Teriparatide is not currently licensed for the treatment of stress fractures and cannot be prescribed outside of the trial. The Ministry of Defence maintains an arrangement for the payment of no-fault compensation to individuals who suffer illness, injury or death as a direct result of participating as a volunteer in research conducted on their behalf and given favourable opinion by the Ministry of Defence Research Ethics Committee (MODREC). As a trial sponsored by an NHS organisation, NHS insurance for negligent harm covers sponsor activities and those of participating NHS trusts. Non-negligent harm does not include side effects, which are listed in the trial protocol and participant information sheet. Under the terms of the no-fault compensation scheme, the Ministry of Defence insures against side effects that are listed in the trial protocol and participant information sheet, as well as unknown side effects that may develop at a later date.

### Outcomes {12}

#### Primary outcome

The primary outcome will be the improvement in radiological healing of lower body stress fractures by two Fredericson grades or more, or reduction in grade to zero, 8 weeks after randomisation. Radiological healing will be assessed using T1 and fluid sensitive (fat suppressed T2W or short tau inversion recovery) MRI sequences and the Fredericson grading system (39, 40). All MRI scans will be assessed independently by two musculoskeletal consultant radiologists blind to the randomisation outcome. Assessors will be notified of any between-assessor grading discrepancies and will be asked to agree a consensus grade.

#### Secondary outcomes

##### Time to radiological healing

Radiological healing will be assessed at weeks 8, 10, 12, 14, 16, 20 and 24 by MRI, with a Fredericson grade zero classified as healed [39, 40]. The time from randomisation to radiological healing will be analysed. No further MRI scans will be necessary when fractures have healed, hence the total number of MRI scans a participant will undergo will vary. All MRI scans will be conducted and graded using the same methodology used for the primary outcome.

##### Time to clinical healing

Clinical healing assessments will commence twice weekly after a participant has been classified as radiologically healed, using a protocol based on the index developed by Beck et al. 2012 [41]. A clinical severity score will be calculated from seven sign and symptom scores: (i) pain during daily activities; (ii) night pain; pain when (iii) running and (iv) hopping; and (v) local tenderness, (vi) pain during percussion, and (vii) local swelling of soft tissue, each at the site of the stress fracture. Clinical assessments will cease when a clinical severity score of zero (i.e. healed) has been achieved. The time from randomisation to clinical healing will be analysed.

##### Time to discharge from Army physical rehabilitation

The time from randomisation to the initial decision to discharge from Army physical rehabilitation will be obtained from participants’ Army medical records. A review by an exercise rehabilitation instructor, physiotherapist and general practitioner will determine when a recruit will be discharged from physical rehabilitation and is, therefore, considered to be fully fit and ready to return to training.

##### Pain symptoms

Participants will rate the level of pain they are feeling on a visual analogue scale once-a-week for the duration of their participation in the study [42, 43]. Pain scores will be analysed as a change from baseline.

##### Health-related quality of life

The Short Form (36) Health Survey (SF-36) will be completed by participants at baseline and every 4 weeks for the duration of their participation in the study to assess their health-related quality of life [44, 45]. The SF-36 is a self-administered 36-item questionnaire that measures health-related quality of life in eight domains (vitality, physical functioning, bodily pain, general health perceptions, physical role functioning, emotional role functioning, social role functioning, and mental health). Each domain is scored from 0 to 100 (lowest to highest level). Physical and mental summary scores will be obtained by aggregating scores across domains and analysed as a change from baseline.

##### Adverse events

Any unfavourable and unintended sign, symptom or illness that develops or worsens during an individual’s participation in the study, and for 4 weeks afterwards, will be recorded and classified as an adverse event (see the “Adverse event reporting and harms {22}” section).

#### Exploratory outcomes

##### Blood and urine biochemistry

Blood and urine samples will be collected at baseline and weeks 4, 8, 12 and 16. Where a fracture remains unhealed at week 16, additional blood and urine samples will be collected at weeks 20 and 24. Venous blood samples will be collected from an antecubital vein. The NHS institution laboratory will analyse blood and urine samples for safety (Table 1). Aliquots of serum, plasma and urine will be stored at − 80 °C, before being defrosted prior to research analyses (Table 1). Analytes will be analysed as a change from baseline. All blood and urine biochemical analyses will be completed by technicians blind to the randomisation outcome.

Slight and transient elevations of serum calcium concentration are normal following the injection of teriparatide. It takes 4–6 h for serum calcium to reach C_max_ and 16–24 h to return to baseline after each dose [24]. Therefore, blood samples will be collected at least 16 h after a participant’s most recent teriparatide injection. Where their regular dosing regimen means treatment would be interrupted to comply with this, the time of their last dose will be recorded. In the event of a hypercalcemia result, a trough measurement will be made within 7 days, before any dose modifications or discontinuations are made. Randomisation will be permitted prior to the receipt of baseline plasma parathyroid hormone concentration. However, in the unlikely event of a participant presenting with a plasma parathyroid hormone concentration outside the adult reference range, they will be called back for review and instructed to stop injecting teriparatide.

##### Bone density and morphology

Bone density will be assessed at the lumbar spine and hips by dual-energy X-ray absorptiometry (DXA) at baseline and week 16 (Lunar iDXA, GE Healthcare, Buckinghamshire, UK). Bone density and morphology will be measured by peripheral quantitative computed tomography (pQCT) scans of the diaphyseal tibia (XCT2000L, Stratec, Pforzheim, Germany), and high-resolution pQCT (HR-pQCT) scans of the metaphyseal tibia and radius (Xtreme CTII, Scanco Medical, Bruttisellen, Switzerland) at baseline and week 16, and week 24 where a fracture remains unhealed at week 16. Bone density and morphology will be analysed as a change from baseline. The non-dominant arm and non-fractured, non-dominant leg will be scanned where possible. A random sample of analysed images (≥ 10%) will be reviewed at the end of the study by an independent assessor blind to the randomisation outcome.

##### Physical activity levels

A wrist-mounted tri-axial accelerometer will be worn by a subgroup of participants for the duration of their participation in the study to assess their physical activity levels. A minimum of 10 participants in each of the control and intervention arms will wear an accelerometer.

##### Long-term future fracture rate

Participants’ Army medical records will be reviewed for 5 years after their final study visit to record any subsequent bone injuries and fractures.

##### Confounding variables

Participants’ alcohol consumption and smoking habits (confounding variables for skeletal health [46]) will be recorded at baseline and every 4 weeks for the duration of their participation in the study. Participants’ physical fitness will be recorded by accessing their Army fitness records.

### Participant timeline {13}

**Fig. 2:**
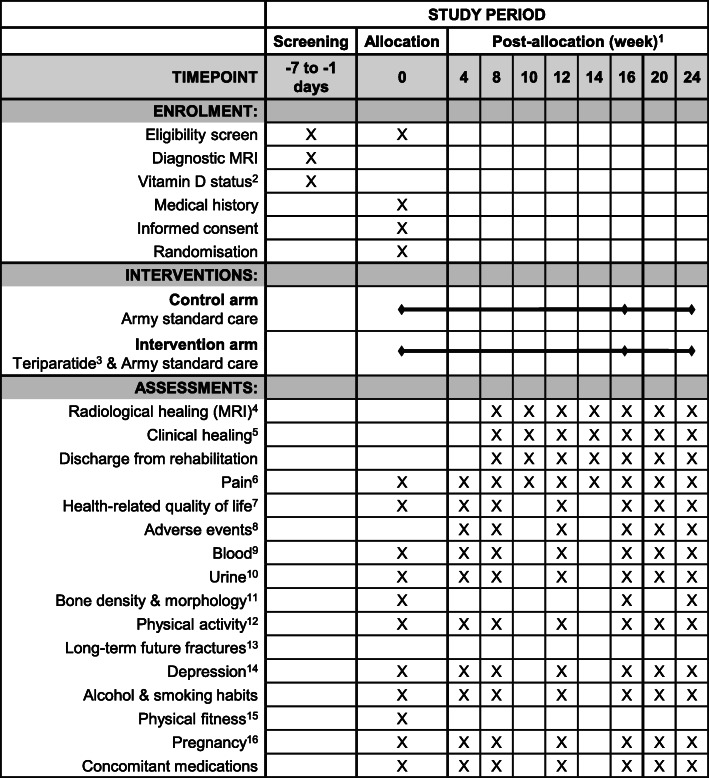
Schedule of study enrolment, interventions and assessments. ^1^Intervention and assessments to continue after week 16 only where fractures are unhealed at week 16; ^2^Serum 25-hydroxyvitamin D < 30 nmol/L, deficient; ≥30 nmol/L, not deficient; ^3^Daily subcutaneous injection (20 μg/day); ^4^Magnetic resonance imaging (MRI) scans to cease when fracture is radiologically healed; ^5^Clinical assessments twice weekly after fracture is radiologically healed, and to cease when fracture is clinically healed; ^6^Weekly visual analogue scale; ^7^Short Form (36) Health Survey; ^8^Adverse events recorded for 4 weeks after participant’s final study visit (i.e. until weeks 20 or 28). ^9^Safety and research blood biochemistry; ^10^Safety and research urine biochemistry; ^11^Dual-energy X-ray absorptiometry (DXA) scan of the lumbar spine and hips (allocation and week 16 only); peripheral quantitative computed tomography (pQCT) scan of the diaphyseal tibia; and high-resolution pQCT (HR-pQCT) scan of the metaphyseal tibia and radius; ^12^Accelerometer worn 24 h/day and replaced four-weekly; ^13^Review of Army medical records for 5 years after participant’s final study visit; ^14^Columbia-Suicide Severity Scale for all at allocation, and four-weekly for participants allocated to the intervention arm; ^15^Review of Army fitness records; ^16^Pregnancy test for all at allocation, week 16 and 24, and four-weekly for participants allocated to the intervention arm

A summary schedule of study enrolment, interventions and assessments is given in Fig. 2.

### Sample size {14}

A total sample size of 136 participants will be required (68 participants per trial arm, prior to accounting for drop-out), based on the primary outcome of an improvement in radiological healing by two Fredericson grades or more, or reduction to grade zero, 8 weeks after randomisation. The sample size was determined using data from Almirol and colleagues’ (2016) 8 week randomised controlled trial in adults aged 21–45 years with recent lower-extremity stress fractures (35). Almirol et al. (2016) reported 67% of participants who received teriparatide achieved a two grades or more improvement in healing or reduction to grade zero (i.e. ‘success’); compared with 43% of participants in the placebo group [35]. Assuming a 20% drop-out rate during the first 8 weeks of the present study, 108 participants (54 participants per trial arm) will complete an MRI scan at week 8; thereby providing 80% statistical power to detect a between group difference (two-sided test, significance level of 5%), if the ‘success’ rate is ≥ 71% in the intervention arm (chi-squared test with continuity adjustment). This equates to an odds ratio of 3.23 or a relative increase in healing of approximately 65%.

### Recruitment {15}

Potential participants with single or multiple lower body stress fractures diagnosed by MRI will be identified by physiotherapists and general practitioners at Infantry Training Centre, Catterick. Potential participants will be given a participant information sheet and will be invited to meet investigators for a detailed explanation of the study as soon as practicable. The planned recruitment period (18 months) has been calculated using the most recent data on rates of lower body stress fracture injuries during Infantry training to enable the study to reach the target sample size.

## Assignment of interventions: Allocation

### Sequence generation {16a}

Eligible participants who provide written, informed consent will be randomised on a 1:1 basis to one of two trial arms using a web-based randomisation process (REDCap). Allocation will be made after eligibility has been confirmed. This process will use blocked randomisation with block lengths of 2–6 in random order. Allocation will be stratified by vitamin D status, measured at baseline (serum 25-hydroxyvitamin D < 30 nmol/L, deficient; or ≥ 30 nmol/L, not deficient), and whether the participant has single or multiple lower body stress fracture sites.

### Concealment mechanism {16b}

Randomisation will be performed centrally using a web-based randomisation process (REDCap).

### Implementation {16c}

The randomisation schedule will be generated by Norwich Clinical Trials Unit (NCTU). Eligible participants will be enrolled by an appropriately trained clinician or delegated member of staff at the NHS institution who will randomise participants using the web-based study database.

## Assignment of interventions: Blinding

### Who will be blinded {17a}

All MRI scans (primary outcome) will be assessed by two musculoskeletal consultant radiologists; all blood and urine biochemical analyses will be completed by technicians; and bone density and morphology images will be reviewed at the end of the study by an independent assessor, all of whom will be blind to the randomisation outcome.

### Procedure for unblinding if needed {17b}

This is an open label design and neither participants nor investigators will be blind to the randomisation outcome. There will be no expected circumstances where the radiologists, technicians or bone density and morphology image assessor will need to be aware of a participant’s allocated intervention.

## Data collection and management

### Plans for assessment and collection of outcomes {18a}

Investigators will be trained centrally in the use of data collection instruments, laboratory equipment, questionnaires, case report forms, and data entry onto a central database. Investigators will follow standard operating procedures and working instructions to help ensure valid and reliable data are collected. Standardising these processes will enhance data quality and reduce bias by detecting and reducing the quantity of missing or incomplete data, inaccuracies, and excessive variability in measurements [47]. Screening and eligibility data will be collected by investigators using case report forms prior to allocation (Fig. 2). Radiological healing will be assessed by two musculoskeletal consultant radiologists blind to the randomisation outcome, using Fredericson grading of MRI [39, 40], and will be recorded using case report forms. Clinical healing will be assessed by investigators using a standardised protocol and case report form [41]. The date of the initial decision to discharge from Army physical rehabilitation will be obtained from participants’ Army medical records and recorded by investigators using case report forms. The SF-36 questionnaire will be completed by participants to assess health-related quality of life [44, 45], and investigators will verbally ask participants the questions in the C-SSRS questionnaire to assess depression. Participants will record their pain on a visual analogue scale [42, 43]. Investigators will ask participants to report adverse events and their use of concomitant medications, which will be recorded using case report forms. Participants’ alcohol consumption and smoking habits will be recorded by investigators completing case report forms. In accordance with standard operating procedures, physical activity levels will be assessed using accelerometers (in a minimum of 10 participants in each of the control and intervention arms), venous blood samples will be collected by investigators, urine samples will be collected by participants, and blood and urine biochemistry will be analysed by technicians blind to the randomisation outcome (Table 1). Pregnancy tests will be conducted according to standard operating procedures. Bone density and morphology will be assessed by investigators using DXA, pQCT and HR-pQCT, according to standard operating procedures, with a random sample of analysed images (≥ 10%) reviewed at the end of the study by an independent assessor blind to the randomisation outcome. Physical fitness will be recorded by investigators accessing participants’ Army fitness records. Army medical records will be reviewed by investigators to record any subsequent bone injuries and fractures.

### Plans to promote participant retention and complete follow-up {18b}

Investigators will maintain regular contact with participants throughout their participation in the trial to offer support as required. Participants will be asked for their preference with regards to the form this will take (i.e. telephone or video calls, text messages, emails, face-to-face consultations). All participants will meet with investigators a minimum of every 4 weeks. Participants who remain onsite at Infantry Training Centre, Catterick will meet with investigators as frequently as every day. Study visits will be scheduled to avoid disruption to each participant’s rehabilitation programme, with the aim to incorporate the trial into routine life within their barracks. Participants who discontinue or deviate from their allocated intervention protocol will remain in the trial for all follow-up visits, provided they are willing. For participants who withdraw or are withdrawn from the study, all data collected up to the date of withdrawal will be retained for use in the study. The reason for withdrawal, if given, and loss to follow-up will be recorded.

### Data management {19}

Each participant will be given a unique trial participant identification number (PIN). Data will be recorded on paper case report forms first and will then be entered under the participant’s PIN onto a central database (REDCap) stored on secure servers at the University of East Anglia. Data will be recorded and entered by investigators (trained for data entry) at Infantry Training Centre, Catterick and the NHS institution. The database will be accessed using unique, individually assigned usernames and passwords, with access limited to study investigators, the Trial Team at NCTU, and external regulators if required. The type of activity a user may undertake will be controlled by the privileges associated with their personal password-protected account and REDCap data access groups. A full audit trail of data changes will be maintained prior to the data lock. Data integrity will be ensured by using referential data rules, and valid value, range, and consistency checks. Staff at NCTU will review case report form data for errors and missing key data points. The trial database will also be programmed to generate reports on errors and error rates.

Servers at the University of East Anglia are protected by firewalls and are patched and maintained according to best practice. The location of the servers is protected physically and environmentally in accordance with the University of East Anglia’s General Information Security Policy 3 (Physical and environmental security). The database and associated code have been developed by NCTU Data Management, in conjunction with the Trial Team. The database software provides several features to help maintain data quality, including maintaining an audit trail, allowing custom validations on all data, allowing users to raise data query requests, and search facilities to identify validation failure/missing data. After completion of the trial, the database will be retained on University of East Anglia servers for on-going analysis of secondary and exploratory outcomes.

### Confidentiality {27}

Paper copies of personal data will be kept at trial sites in a secure location with restricted access. Following consent, identifiable data will be kept on the trial database to allow investigators to contact participants in order to arrange study visits. Only authorised Trial Team members will have password access to this part of the database, and this information will be destroyed one month after closure of the study. The confidentiality of participants’ personal data will be ensured by not recording participant names on case report forms and limiting access to personal information held on the NCTU database. The PIN will be the primary identifier for a participant, with secondary identifiers of their date of birth and initials. The participant’s consent form will carry their name and signature and will not be kept with any additional participant data. The identification, screening and enrolment logs, linking participant identifiable data to the pseudoanonymised PIN, will be held locally and securely by trial sites. Trial records will be archived by Norfolk and Norwich University Hospitals NHS Foundation Trust (NNUH) as per current regulatory requirements. Data will be handled in accordance with provisions of the General Data Protection Regulation (EU) 2016/679 and the Data Protection Act 2018.

### Plans for collection, laboratory evaluation and storage of biological specimens for genetic or molecular analysis in this trial/future use {33}

No specimens will be collected for genetic or molecular analysis.

## Statistical methods

### Statistical methods for primary and secondary outcomes {20a}

A full statistical analysis plan (SAP) will be developed by the Trial Statistician and Chief Investigator before the final data lock and will be submitted to the independent Data Monitoring Committee (DMC) and Trial Steering Committee (TSC) for approval [48]. The primary efficacy analysis will be based on the intention-to-treat principle in which all participants will be analysed according to the trial arm to which they were allocated, regardless of compliance. The primary outcome (radiological healing by two Fredericson grades or more, or reduction in grade to zero, 8 weeks after randomisation) will be analysed using a logistic regression model. This model will include the baseline stratification variables (vitamin D status and single or multiple lower body stress fractures); anatomical site of fracture; baseline MRI grade; allocated trial arm; and any other baseline prognostic variables pre-specified in the SAP. The estimate of efficacy will be the between group difference from the analytical model, calculated with 95% confidence intervals and with statistical significance set at the 5% level (two-sided). Secondary outcomes will be analysed in an analogous manner, i.e. using appropriate statistical models with stratification variables, additional specific prognostic variables (including the baseline value of the outcome where available) and allocated trial arm. A secondary analysis of MRI grading at each time point using a logistic regression model with a generalised estimating equation approach to parameter estimation and a specified auto-regressive correlation structure, i.e. to allow for the correlation in outcome between timepoints, will also be completed.

### Interim analyses {21b}

No formal interim efficacy analyses are planned. Recruitment and withdrawal rates will be reviewed during the trial on an ongoing basis. The independent DMC will review information on the progress and accruing data of the trial (e.g. recruitment rates, data quality, adherence to protocol treatment and follow-up, main study outcomes, and safety data) and provide advice on the conduct of the trial to the independent TSC. The TSC, on recommendation from the DMC, will have the ability to propose protocol changes, modify target recruitment, halt recruitment within a subgroup, and in exceptional circumstances prematurely terminate the trial. The Ministry of Defence will also have the ability to terminate the trial if, for example, it fails to deliver the required recruitment rate.

### Methods for additional analyses (e.g. subgroup analyses) {20b}

At present, no sub-group analyses are planned. Any that arise will be pre-specified in the SAP.

### Methods in analysis to handle protocol non-adherence and any statistical methods to handle missing data {20c}

The primary efficacy analysis will be based on the intention-to-treat principle in which all participants will be analysed according to the trial arm to which they were allocated, regardless of compliance. A secondary, sensitivity efficacy analysis will be carried out using imputation of missing data, if deemed appropriate based upon likely mechanism of ‘missingness’ and the degree of missing data. This will be specified in the SAP. The quantity of missing data will be reported, and a comparison made of those included in the primary analysis and those excluded due to missing data (either missing the primary outcome or baseline data for the analytical model).

### Plans to give access to the full protocol, participant level-data and statistical code {31c}

Requests for access to the full protocol, data, and statistical code will be considered, and approved in writing where appropriate, after formal application to the TMG or TSC. After completion of the trial, applications can be made to NCTU.

## Oversight and monitoring

### Composition of the coordinating centre and trial steering committee {5d}

#### Trial Team

The Chief Investigator, Trial Manager and the NCTU will be responsible for the day-to-day running of the trial and implementing trial-specific processes. They will work together with representatives from the Ministry of Defence as the Trial Team.

#### Trial Management Group

The TMG (Sponsor Representative, Chief Investigator, and Ministry of Defence, NCTU and University of East Anglia co-investigators) will assist with developing the design, coordination and strategic management of the trial.

#### Trial Steering Committee

The TSC will be the independent group responsible for oversight of the trial and ensuring it is performed according to Good Clinical Practice in order to safeguard participants. The TSC will provide advice to the Chief Investigator, NCTU, the funder (Ministry of Defence) and Sponsor (NNUH) on all aspects of the trial through its independent chair.

### Composition of the data monitoring committee, its role and reporting structure {21a}

#### Data Monitoring Committee

The DMC will be independent from the Sponsor and competing interests and will be the only oversight body that has access to accumulating comparative data. The DMC will be responsible for safeguarding the interests of participants, monitoring the accumulating data, and making recommendations to the TSC on whether the trial should continue as planned. The DMC will consider data in accordance with the SAP and will advise the TSC through its chair.

### Adverse event reporting and harms {22}

The definitions of harm according to the European Union directive 2001/20/EC Article 2 and detailed guidance laid out in 2011/C172/01, based on the principles of the International Council for Harmonisation for Good Clinical Practice, will be applied in the RETURN trial. All adverse events and reactions will be assessed for seriousness, causality, severity and expectedness and will be reported to the relevant regulatory bodies. Any unfavourable and unintended sign, symptom or illness that develops or worsens during the study (and for 4 weeks after a participant’s final visit) will be classified as an adverse event, whether or not it was considered to be related to the study intervention. Adverse events will include unwanted side effects, sensitivity reactions, abnormal laboratory results, injuries and inter-current illnesses, and may be expected or unexpected. All events will be followed until resolution. There will be no exempted adverse events in this trial. A notifiable event will be grade 3 (or higher) haematological toxicity (according to Common Terminology Criteria for Adverse Events v5.0), identified in blood tests whilst a participant is taking trial medication. The TMG will review line listings of cumulative serious adverse events. Any concerns about potential emerging toxicity will be escalated to the independent DMC. The DMC will review safety data including reported frequencies of non-serious adverse events, serious adverse reactions and suspected unexpected serious adverse reactions.

### Frequency and plans for auditing trial conduct {23}

The frequency, type and intensity of routine and triggered on-site monitoring will be detailed in the quality management and monitoring plan. On-site visits will be arranged every 6 months, or more frequently if concerns are raised by the Trial Team, Sponsor or TMG. Site monitoring will be performed by a member of NCTU delegated to perform monitoring tasks. The quality management and monitoring plan will also detail the procedures for review and sign-off of monitoring reports.

### Plans for communicating important protocol amendments to relevant parties (e.g. trial participants, ethical committees) {25}

Amendments to the protocol will be agreed by the TMG and submitted to MODREC for approval. Amendments will not be implemented until approvals from the Medicines and Healthcare products Regulatory Agency (MHRA) and Health Research Authority have also been received, and research sites have confirmed their acceptance with NCTU. The public study record on ClinicalTrials.gov will be updated with protocol amendments.

### Dissemination plans {31a}

The results of the trial will be disseminated regardless of the direction of effect and will be reported according to the Consolidated Standards of Reporting Trials statement [49]. Results of the trial will be disseminated by publication in appropriate journals and conference presentations. Permission to publish from the Ministry of Defence will be required prior to publication. A lay summary of the trial results will be given to all participants at the end of the study.

## Discussion

The RETURN trial will be the first study to investigate the effect of teriparatide on lower body stress fracture healing in young, otherwise healthy men and women. The use of MRI and blinded review using the validated Fredericson stress fracture classification system will enable radiological healing to be examined as the primary and as a secondary study outcome [39, 40]. As the gold standard for assessment of bone stress injury, MRI has been shown to be the most sensitive and specific imaging test for diagnosing lower body stress fractures [50] and will, therefore, allow injury severity and recovery progress to be closely monitored. In addition, MRI is non-invasive and will not expose participants to ionising radiation, in contrast to conventional radiology, nuclear scintigraphy and computed tomography [50]. The protocol of two-weekly MRI scans from weeks 8–16, and four-weekly from weeks 16–24, until fracture healing, will enable the time to radiological healing to be captured with greater accuracy than in previously published literature [27–30, 32, 33, 35]. By using MRI to assess healing, the efficacy of teriparatide will be examined using an outcome that participants and reviewers are unable to manipulate.

The recovery of participants’ physical function and health will be assessed using a variety of methods. Using a clinical severity score to assess clinical healing once a fracture is radiologically healed will enable a standardised assessment of physical function to be made [41]. In addition, the time to discharge from physical rehabilitation will provide a measure of physical function and readiness to return to training. The time to discharge from physical rehabilitation will be affected by confounding variables e.g. (i) the physical demands of the training course the participant is re-joining, determined by the week of training at the time of injury, and the physical intensity of their regiment’s training programme; and (ii) the participant’s compliance to, and engagement with, their rehabilitation programme. Although reliant on self-report, the pain visual analogue scale and SF-36 are relatively easy to complete and will add minimal additional burden to participants. The visual analogue scale has been shown to be a valid and reliable tool to assess pain [42, 43] and will be interpreted alongside the participant’s record of analgesic use. The widely used SF-36 will provide a valid and reliable measure of health-related quality of life, functional health and wellbeing [44, 45]. The inclusion of assessments of blood and urine biochemistry; bone density and morphology; and physical activity levels will provide an insight into the mechanisms underlying the hypothesised beneficial effect of teriparatide on stress fracture healing. Participants’ alcohol consumption, smoking habits and physical fitness (confounding factors for skeletal health [46]) will be recorded so the possible effect of these variables on study outcomes can be analysed.

One of the challenges associated with the collection of the outcome measures is the burden placed on participants. To mitigate trial burden, study visits will be scheduled to avoid disruption to participants’ Army training and physical rehabilitation programme, and with transport provided as required. In addition, the week 4 study visit, when many participants are expected to be on sick leave, will be completed at the participant’s home if required. The requirement for participants to be undergoing Phase One or Two Army training, and focus on Infantry training in particular, means a standardised rehabilitation programme will be followed by all participants. This will enable the efficacy of teriparatide to be examined with greater control over confounding variables, such as variation in rehabilitation programmes and access to facilities, than would likely be possible among athletic or civilian cohorts.

### Safety

One safety concern associated with teriparatide during its development was the possible association with osteosarcoma, due to a preclinical animal study reporting a dose-dependent increase in the incidence of osteosarcoma and some other rare bone tumours in one breed of rat, treated with near life time, high doses of teriparatide [25]. This finding was not observed in subsequent animal studies with other animal models or breeds of rat, and treatment with teriparatide in humans has been demonstrated to be safe, with no association between teriparatide and osteosarcoma observed since surveillance studies commenced in 2003 and 2009 [24–26]. The maximum total duration of treatment with teriparatide was increased from 18 to 24 months by the European Medicines Agency and US Food and Drug Administration based on safety data and post-marketing experience [51, 52].

For safety, participants’ blood and urine samples, collected at baseline and every 4 weeks throughout the study, will be analysed to ensure they are within adult reference ranges (analytes listed in Table 1). Hypercalcaemia and hypercholesterolaemia have been reported in individuals treated with teriparatide [24]; hence, serum calcium and total cholesterol will be monitored throughout the study. Teriparatide may cause small increases in urinary calcium excretion [24]; thus, participants’ urine calcium will be monitored. Participants will be expected to be otherwise healthy because in order to be undergoing Army training, they will have recently passed a physician screened medical assessment.

Teriparatide should not be given to individuals with open epiphyses [24]; thus, potential participants who present with open epiphyses in their diagnostic MRI scan will be excluded from participating. Signs of depression will be monitored using the C-SSRS because depression is a possible side effect of treatment with teriparatide [24]. Women will require a negative serum pregnancy test result to be eligible to participate and must agree to use of a highly effective form of contraception (failure rate < 1% per year, e.g. combined oestrogen and progestogen, or progestogen-only hormonal contraception, or intrauterine device) because teriparatide is contraindicated for use during pregnancy [24]. Treatment with teriparatide will be discontinued for participants who become depressed or pregnant. A negative urine pregnancy test result will be required prior to teriparatide re-dispensing. Dual-energy X-ray absorptiometry, pQCT, and HR-pQCT scans will expose participants to ionising radiation, however, the total radiation exposure for each participant will be relatively low, categorised as minor risk (0.1–1 mSv; Category IIa) [53]. A negative pregnancy test result will be required before DXA, pQCT, and HR-pQCT scans to avoid exposing pregnant women to ionising radiation.

### Protocol amendments

The following amendments were approved by MODREC, resulting in the current study protocol described here (version 1.6). In June 2019, during the planning stage, the study was changed from a double blind, placebo-controlled trial to an open label design, with usual Army standard care as the control arm. The principal reason for this change was the rejection of return to training as a potential primary endpoint, since readiness to return to training was considered too subjective, and the subsequent choice of an objective primary endpoint of improvement in radiological healing assessed by MRI. There is no known biological mechanism by which participants can influence radiological measures of stress fracture healing, therefore, this objective endpoint lends itself to an open label design. A second reason was the continued unavailability of a suitable placebo. An open label design has the added benefits of reducing the burden on control arm participants by removing the requirement for them to administer daily injections of placebo for 24 weeks, and avoids the possible ethical implications of requiring them to do so; it may also increase compliance within the intervention arm since participants will know they are receiving active drug. The practical issue of adherence to the injection regime by military recruits, knowing they are receiving drug, is important for the future roll out of any treatment regime. Additional amendments were the protocol for calcium and vitamin D supplementation where serum calcium is < 2 mmol/L; the requirement for participants to be prescribed vitamin D as part of their standard care (if vitamin D deficient); MRI scans at weeks 20 and 24, where a fracture remains unhealed at week 16; a subgroup of participants to wear accelerometers to assess their physical activity levels; and clarification regarding randomisation whereby allocation will be stratified by vitamin D status and single or multiple lower body stress fracture sites.

In August 2019, during the study planning stage, the exclusion criteria were modified to exclude potential participants with depression, indicated by the C-SSRS, and to add the provision that participants who exhibit signs of depression (as indicated by the C-SSRS) will have their teriparatide treatment discontinued.

In April 2020, an inclusion criterion was reworded from ‘lower limb stress fracture’ to ‘lower body stress fracture’, where lower body includes the pelvic girdle, sacrum, coccyx and lower limb. The pQCT scanning of the diaphyseal tibia was added because the available HR-pQCT scanner (Xtreme CTII, Scanco Medical) is unable to analyse this anatomical site. As potential confounding variables, the collection of participants’ alcohol consumption, smoking habits and physical fitness were added to the protocol. Analysis of butyrate was added as an exploratory outcome due to emerging evidence that it may influence parathyroid hormone-dependent bone formation. Butyrate produced by gut luminal microbiota has a role in triggering regulatory pathways which are required for the anabolic action of parathyroid hormone in bone [54]. Study MRI results are considered by the rehabilitation staff to constitute additional clinical information relevant to participants’ treatment and discharge from rehabilitation. Therefore, once the decision to discharge a participant from rehabilitation and to return to training has been made and recorded, it was clarified that the most recent MRI scan results will be released to the rehabilitation staff who will then be at liberty to proceed or to revise their decision as they see fit, before communicating the outcome to the participant. This change was designed to fulfil a duty of care to participants, but without interfering with usual Army standard care or the initial decision to discharge from rehabilitation (a secondary outcome).

In October 2020, in response to the COVID-19 pandemic, the option for some NHS site study visits and procedures to be completed remotely (via telephone or video call) or at Infantry Training Centre, Catterick with NHS oversight, were added to the protocol. These include the option for adverse event and concomitant medication checks to be completed by NHS staff via telephone or video calls; and the option for blood and urine samples to be collected at Infantry Training Centre, Catterick with NHS oversight. Allocation, confirmation of eligibility and obtaining informed consent will remain the responsibility of the NHS institution, but can be completed by telephone or video call. If access to the NHS hospital is restricted or MRI services are unavailable, MRI scans can be completed using a scanner local to Catterick instead of those at NHS sites. The objectives behind these COVID-19 changes were to protect participants and investigators from SARS-CoV-2 and the development of COVID-19, by eliminating as far as possible the need to visit NHS hospitals and by following local and national government restrictions on social distancing and travel; to remove the greatest burden possible from NHS staff, in line with MHRA and National Institute for Health Research guidance (published 21st May 2020 [55]); and to keep the trial open, if safe to do so, in the event of future virus outbreaks. It was clarified that women will require a negative pregnancy test result before teriparatide dispensing, and before DXA, pQCT, and HR-pQCT scans. A formal review of the recruitment rate and feasibility of the study by the TMG (originally scheduled to be evaluated 6 months after the trial opening) will take place 6 months after the trial reopening following the pause in recruitment caused by the COVID-19 pandemic.

### Perspectives and future directions

If the study finds teriparatide to be effective for accelerating stress fracture healing, military recruits injured with lower body stress fractures could be treated with teriparatide as part of their standard care, subject to balance of investment and health economics analysis, approval by the Army Drugs and Therapeutics Committee, and incorporation into the Army stress fracture treatment protocol. The long-term future fracture rate recorded in the study will provide an insight into the fracture preventative potential of teriparatide in young, otherwise healthy adults. Investigating the efficacy of teriparatide administered using an alternative delivery route that would eliminate the need for daily subcutaneous injections would be of interest. Oral dosing is potentially a more convenient method of administering teriparatide [56]; however, it is currently being investigated in early phase clinical trials and was not available for the present study.

## Trial status

The current protocol is version 1.6 (10th September 2020). The first participant was recruited on 29th January 2020. Recruitment was paused on 24th March 2020 due to the COVID-19 pandemic. No participants had reached the week 8 primary endpoint by this date. The TMG made the decision on 24th March 2020 that no further in person assessments would be undertaken, but where possible participants would complete secondary endpoint assessments by telephone, up to the week 8 timepoint, before being withdrawn from the study. The first participant after the pause in recruitment caused by the COVID-19 pandemic was recruited on 2nd October 2020. Recruitment will continue until approximately April 2022. The incidence of stress fractures and subsequent recruitment has been lower than anticipated due to the COVID-19 pandemic. Therefore, the TMG intend to obtain approval to expand recruitment from Army recruits to the wider UK Armed Forces so the required recruitment rate can be met.

## Data Availability

Requests for access to data will be considered, and approved in writing where appropriate, after formal application to the TMG or TSC. After completion of the trial, applications can be made to NCTU. Ministry of Defence approval will be sought by NCTU.

## Abbreviations

C_max_: Maximum concentration
C-SSRS: Columbia-Suicide Severity Rating Scale
DMC: Data Monitoring Committee
DXA: Dual-energy X-ray absorptiometry
HR-pQCT: High-resolution peripheral quantitative computed tomography
MHRA: Medicines and Healthcare products Regulatory Agency
MODREC: Ministry of Defence Research Ethics Committee
MRI: Magnetic resonance imaging
NCTU: Norwich Clinical Trials Unit
NHS: National Health Service
NNUH: Norfolk and Norwich University Hospitals NHS Foundation Trust
PIN: Participant identification number
pQCT: Peripheral quantitative computed tomography
RETURN: Research on Efficacy of Teriparatide Use in the Return of recruits to Normal duty
SAP: Statistical analysis plan
SF-36: Short Form (36) Health Survey
TMG: Trial Management Group
TSC: Trial Steering Committee
